# Mapping the ethical aspects in end-of-life care for persons with a severe and persistent mental illness: A scoping review of the literature

**DOI:** 10.3389/fpsyt.2023.1094038

**Published:** 2023-03-16

**Authors:** Loïc Moureau, Monica Verhofstadt, Axel Liégeois

**Affiliations:** ^1^Faculty of Theology and Religious Studies, KU Leuven, Leuven, Belgium; ^2^Medical and Health Sciences, Ghent University, Ghent, East Flanders, Belgium

**Keywords:** ethics, SPMI, values, end-of-life care, scoping review

## Abstract

Persons with severe and persistent mental illness (SPMI) make up a vulnerable group within mental healthcare and society. Not only do they suffer from long-term, serious psychiatric disorders; they often also experience considerable problems in their psychosocial functioning. Research has disclosed that the care needs of this target group are complex, and that the life expectancy of these persons is significantly lower than in the general population. Given (1) the lower life expectancy of persons with SPMI, (2) the higher suicide risk related to mental disorders, and (3) the legalization and practice of medical assistance in dying in an increasing number of countries, it is of utmost importance to map the ethical aspects and challenges of end-of-life care needs in persons with SPMI. Therefore, we charted the way end-of-life care is provided for them by means of a scoping review of the scientific literature, with an emphasis on the ethical aspects surrounding it. We explore existing ethical dilemmas; the underlying ethical values, principles and attitudes; and the locus and stakeholders of ethical dialog regarding end-of-life care in persons with SPMI. The results indicate that the four guiding principles of biomedical ethics can well be identified in the literature, and are each addressed in their own specific way: Autonomy in relation to questions regarding the decision-making capacity of persons with SPMI; Justice in relation to access to quality care and the presence of stigma; and Non-maleficence and Beneficence in relation to the ongoing debate regarding the benefits and obstacles in applying palliative care approaches in the context of psychiatry, and the status of the futility-concept therein. Personal virtues and attitudes in care professionals, like compassion, non-abandonment and upholding dignity are key, as care professionals are the main advocates of persons with SPMI, which often lack an extensive social network. Further, we find that the ethical dialog is mainly focused on care professionals and relatives, rather than the persons with SPMI themselves. This is reflected in the existing research that often had the voices of the latter missing. Future research may benefit from the inclusion of persons with SMPI’s first-hand accounts. End-of-life care for persons with SPMI may benefit from identifying and integrating (locally developed) good practices like cross-sectoral education, specific care models, and ethics support.

## Introduction

For the concept “severe and persistent mental illness” (SPMI) no consensus definition exists in the scientific literature. A study by Zumstein and Riese shows that authors who publish about this target group do formulate three important common characteristics: (1) the persons suffer from one or more psychiatric disorders, as formulated in DSM 5; (2) the course of the disorder is chronic; and (3) results in serious limitations in their psychosocial functioning (1).

Research has repeatedly shown that life expectancy is significantly lower, up to 10–20 years compared to the general population (2–5). The causes are manifold: a higher risk of suicide; the side effects of long-term use of psychotropic medication; obesity, smoking and general poor lifestyle; late detection of cancer and other serious conditions, due to care-avoiding behavior and difficulties in communication. This, combined with other issues such as limited social network, the struggle with serious illness, drug abuse, risk of homelessness, stigma and self-stigma make people with SPMI a group with considerable increased vulnerability (6).

The high level of complexity of actual care needs, combined with the lower life expectancy and great suffering that persons with SPMI face, suggests that end-of-life (EOL) care for these individuals also involves a high level of complexity. This may often involve various ethical dilemmas and the weighing up of ethical values, especially given the increased vulnerability of this target group. An ethical approach to care is primarily concerned with the question of how to achieve “good care,” or at least the “best possible care,” with the aim of ensuring that all parties involved in that care are treated with dignity. In this study, we examine the main dilemmas, values and actors in the ethical dialog in the context of end-of-life care for persons with SPMI. Given that this is a new topic within a broad research field, we opted for a scoping-review method in order to expose the main outlines of the theme and the challenges surrounding it (7, 8).

This review thus is related to, but also distinct from studies that have been done on the ethical aspects of medically assisted dying, e.g., euthanasia, in the context of severe psychiatric suffering, on the one hand [e.g., (9, 10)], and studies that shed light on the issue of life-sustaining and palliative care for persons with S(P)MI, on the other [e.g., (2, 5, 11)]. This review is wider than those mentioned, as it either uses a broader definition of end-of-life care, or focuses specifically on ethical aspects (Annex 1).

## Research questions

In this scoping review of the scientific literature, we aim to answer the following three research questions – which serve as inclusion criteria content-wise:

(1) What ethical dilemmas and issues arise in end-of-life care for persons with SPMI?
(2) What values, principles, virtues, and attitudes are used to describe and frame these issues?
(3) “How does ethical reflection and dialog regarding these issues take place in practice and who participate in this?

## Methodology

For this research we conducted the first scoping review of the literature available in 2000 – June 2021, using selection criteria based on the PRISMA-method as described below. We searched using the combination of search terms: [(“severe and persistent mental illness”) AND (“end of life” OR “palliative” OR “euthanasia” OR “medical assistance in dying” OR “suicide” OR “advance care planning”) AND (ethics OR moral OR values)] in the databases Pubmed, Pubmed Central, Medline, Scopus, Psycharticles, and ATLA. Only peer reviewed articles published in English or in Dutch were included. Two authors, LM and AL, independently reviewed title and abstract to select relevant studies that met the inclusion criteria. In case of disagreement, a response group was consulted each time and decisions were made in consensus. No additional quality check was performed.

## Results

A total of 844 articles were identified through the search, 12 sources were added based on the authors’ general knowledge of the literature. The 856 sources are reported in the PRISMA flow diagram (Annex 2) (12). After removing duplicate results, we came up with 310 results that were screened by title and abstract. 255 titles were excluded because they were not relevant to the study – not addressing the research questions. After reviewing the full text, another five articles were not retained because they did not meet the content requirements for inclusion. Thus, a total of 50 articles were withheld from the study; including various types of literature reviews, case studies, qualitative studies, surveys, opinions and original research. The studies are also geographically dispersed, with data coming from countries such as Australia, Switzerland, the United States, the Netherlands, Belgium, and India (Annex 3).

The articles were then inductively coded, based on the research questions. In a next step, five central themes were identified, three of which are related to research question 1, and the other two to research question 2 and 3: (1) autonomy and decision-making capacity; (2) equity and access to care; (3) the desirability of a palliative care approach in psychiatry and the concept of “futility”; (4) the importance of virtues and attitudes; (5) stakeholders in reflection and dialog.

The findings of the study are largely structured using the four principles of biomedical ethics, as formulated by Beauchamp and Childress (13). Although no deductive framework was used in analyzing the content of the sources, we observed that many of the cited articles in this review explicitly refer to these four principles – sometimes only discussing one or two, sometimes adding additional issues. As authors, we therefore chose to adhere to this well-known, ethical structure where applicable.

### Autonomy and decision-making capacity

Autonomy is a central value in Western society. Also in healthcare, making one’s own choices based on sound information is a fundamental ethical principle. In the care of persons with SPMI in general, and especially in the context of the end of life, this value is often under debate. Tensions between only respecting “pure” patient-autonomy on the one hand and providing optimal care and preventing harm on the other often arise. Many authors mention the importance, but also the many difficulties and thresholds regarding assessing and respecting the choices of persons with SPMI and their judgement.

The underlying causes are diverse. A first element is the pathology of these persons: is the choice authentic, or prompted by the clinical picture (14, 15)? Further, by receiving care for a long time, the question can also be whether these persons are not too accustomed to being dependent on care, or vice versa: that “not wanting to depend on care” fundamentally influences the choice of the person with SPMI in choosing, e.g., physician-assisted suicide or euthanasia (16). Communication difficulties and the perceptions of care professionals form a barrier as well. Lastly, sometimes care professionals are afraid to discuss the end of life (17) or they start from the perception of vulnerability, which does not always correspond to reality and might lead to disproportional use of paternalism and coercion (18).

In the care of persons with SPMI, the classic dilemma between respecting autonomy on the one hand and a paternalistic approach on the other comes back to haunt us: does the care professional follow the patient’s choice; or does he or she act out of a desire to do justice to the non-maleficence principle and offer optimal care? With regard to cases specifically influencing standard medical end-of-life care, we find various examples in the literature of persons with mental disorders who are not compliant, refuse to take (pain) medication and refuse dialysis or life-extending treatments – severely complicating the application of good palliative care in some cases (2). Care professionals are sometimes faced with the difficult choice of shaping (end-of-life) care not on the basis of voluntariness but within a framework of coercion (19).

The drafting of documents on advance care planning and directives, as well as their implementation, is also subject to discussion (19–21). In the literature, the views of care workers on the subject of participation of people with SPMI in their own end-of-life decision making are divided: psychiatrists and other care workers are primarily trained to prevent suicide and to act in a life-saving manner. Especially in the context of end-of-life decisions however, this life-sustaining attitude comes under pressure (22, 23). This is particularly true in those contexts where a legal framework regarding euthanasia or physician assisted suicide exists. The personal values of care professionals come under pressure in those paradoxical situations where both the prevention of suicide, and supporting patients in their wish for physician assisted dying are part of the care-giving process (10, 24).

We also find various recommendations in the literature to address these dilemmas. For example, it is argued that the wishes and choices of individuals should be taken seriously (25, 26). Several authors underline the fact that persons with SPMI can express their wishes and desires, and are entitled to additional support in doing so (competent supported decision-making) (16). Furthermore, the use of coercion has a negative impact on all stakeholders (27). In some cases, it is also important to involve surrogate decision-makers (2, 20, 21). These can be the family, although family members are often “absent” with this target group (11, 28, 29). Research shows, however, that representatives and patients do, by and large, have the same vision on care (30).

There is a need for more research, guidelines and legal clarity on decision-making capacity (14, 16, 31). For some authors, decisions and clarification of decision-making capacity is clearly framed as an ethical consideration (alongside the legal and medical) (2). If, counseling would succeed in dealing with the fluctuating nature of decision-making competence (both in time and in content) in an adequate and open manner; sufficiently taken into account the fact that many decisions are not made purely on the basis of a rational consideration, but also on the basis of emotions and personal values and goals; and sufficiently focus on shared decision-making, this could be a great benefit for this target group as well (16, 23, 32).

### Equity and access to care

Perhaps the greatest ethical challenge regarding good end-of-life care for persons with SPMI that we find in the literature concerns the value of justice, concretized in the theme of access to care. Persons with SPMI have complex care needs. Access to quality care, adapted to the specific needs of these persons, is a challenge in itself. This seems even more true when we zoom in on good end-of-life care, where several specific problems and concerns arise.

In the literature, we find from various authors that access to quality palliative care in general, and forms of active termination of life (e.g., euthanasia or physician-assisted suicide) in specific, is limited for persons with SPMI, for various reasons. Although the end-of-life wishes of these persons are the same as in the general population, life expectancy is much lower and the needs for good care are higher, the access to quality care is significantly lower (2, 3, 33). The causes of this are again very diverse, but doubts about decision-making capacity, the absence of written directives and representatives, and difficulties in communication are at least the first cluster.

Various authors also point to the fact that the care landscape in this area is very fragmented and “siloed”: somatic care and mental health care often work separately from one another (3, 11, 19, 27, 29, 34–36). Coordination between different actors in the care is often difficult. Persons with SPMI with a life-threatening condition therefore risk falling between two stools and becoming victims of stigma. In sum, there is fragmentation instead of integration of care.

There are several other reasons, stated in the literature, for the difficult access to quality end-of-life care, e.g., late detection of severe somatic conditions, different perception and expression of pain by persons with SPMI, lack of health insurance, limited insight into illness, refusal of medication and previous negative experiences by persons with SPMI with (institutionalized) care (2, 3, 5, 37–39).

In addition, some persons with SPMI display disruptive behavior (including verbal and physical aggression) due to their mental illness. This can be an additional obstacle to quality palliative care. More specifically, several authors mention drug seeking behavior, especially in persons with addiction problems (29, 40). This makes the correct use of pain-medication more difficult.

The lack of a good network and family can also be a reason why persons with SPMI have insufficient access to care. Prejudice on the part of the care professionals themselves and self-stigma on the part of the person seeking care, e.g., regarding drug seeking behavior, also contribute to the problem (19, 28, 29, 41, 42).

The unequal (even unjust?) treatment of this target group also continues in the field of scientific research. Here, too, persons with SPMI are often excluded because of (perceptions regarding) their behavior, more difficult communication or doubts about their decision-making capacity (18, 34, 35, 43). This certainly applies to qualitative research, although authors indicate that allowing the voice of the person with SPMI to be heard is essential (44). Exclusion from scientific research reinforces the inequality between this group and the rest of the population.

As mentioned before, a specific component within this theme concerns specific forms of types of care around the end of life. The use of, e.g., palliative sedation and DNR, and legal access to euthanasia and assisted suicide are in itself controversial issues within the health care sector. Certainly within mental health care, there are many questions as to whether these are morally and legally permissible, and under what conditions. Concerning medical assistance in dying for patients with a psychiatric disorder, there are many points of discussion regarding, again, the assessment of mental competence, the sustainability of the request to end life, the impact and non-alleviability of suffering, the irremediableness of the mental disorder, the position of relatives, etc. (9, 45). In the literature specifically mentioning SPMI, regarding to some authors, all these issues are pressing, because of their vulnerability, reduced access to quality care and significant suffering (10, 31, 46–49).

The literature offers the care sector various solutions and good practices for dealing with this difficult issue. First of all, it points to the responsibility of the government and care organizations to invest more in care, in terms of staff, resources and training (37). The stronger integration of care and the breaking down of the walls between the different care sectors is paramount. In addition, it can be very valuable to invest in training that enables care professionals to properly conduct end-of-life conversations with their SPMI-patients (34, 35, 50). Cross-training between mental health and palliative care professionals may also be advisable (17). Dealing correctly with disruptive behavior, building mutual trust and the therapeutic alliance, and allowing sufficient room for creativity can all be threshold factors. Further, there are several good practices already in place, such as community-based models, and the development of local palliative care initiatives close to where the persons with SPMI are living – rather than a classic, institutionalized forms of care (29). Often persons with SPMI also suffer from homelessness, and there is a great distrust of “traditional” health care, with which several negative experiences (e.g., coercion) have been built up. Access to palliative care consultations within institutionalized mental health care could also lower the threshold for all stakeholders (51).

### The desirability of a palliative care approach in psychiatry and the concept of “futility”

The scientific literature clearly indicates that there is a growing need for care provision that truly meets the needs of persons with SPMI. A model of care that borrows elements from palliative care, “a palliative care approach,” is put forward by many authors (37, 49, 52–54). This approach is very much linked to a consideration of two central principles in bioethics: non-maleficence and beneficence.

Trachsel et al. (55) proposed the following definition of Palliative Psychiatry: “Palliative psychiatry (PP) is an approach that improves the quality of life of patients and their families in facing the problems associated with life-threatening severe persistent mental illness (SPMI) through the prevention and relief of suffering by means of a timely assessment and treatment of associated physical, mental, social, and spiritual needs. PP focuses on harm reduction and on avoidance of burdensome psychiatric interventions with questionable impact.”

Central to the debate is the concept of “futility.” In medicine, futility can be defined in several ways, but can, regarding to some authors, “be determined by the likelihood of achieving a target state, or by the initial condition of the patient, as interventions are considered” (25). The concept is mainly used within somatic care and is relatively unknown in the world of psychiatry. In fact, the concept seems to evoke a lot of resistance, because it could be linked to the “giving up” of the patient and the loss of hope. The same applies to the concept of “palliative care,” which, despite the broad definition by the WHO, still retains the connotation of dying (52).

Nevertheless, several authors point out that careful handling of the concept of futility and palliative care – outside the context of the dying process – could have many advantages for the care of persons with SPMI (25, 43, 56). Authors point out the disadvantages of therapeutic tenacity and the pure emphasis on recovery from a medical point of view – such as the side effects of psychotropic medication or electroshock therapy – and the advantages of focusing primarily on quality of life and a holistic approach. These authors argue that palliative care does not have to exclude recovery, but rather makes it possible to focus on a meaningful way of life, the development of trusting relationships and creativity in the approach to care (36, 37, 53–55). The debate regarding the desirability of a palliative care approach center thus on dilemmas regarding doing good (beneficence) and causing no harm (non-maleficence).

Although many authors refer to this palliative care approach, more research and further development of the concept appears to be important. For example, this rather conceptual approach has yet to be translated into concrete practice. A good example from practice seems to be “oyster care”: an approach that uses the metaphor of the oyster’s protective exoskeleton to shape palliative care in practice: a dynamic and safe care environment, with great emphasis on the holistic approach – especially the somatic and existential aspects of care. Quality of life and the control and alleviation of symptoms are paramount (37, 48).

Furthermore, more attention should be paid to “staging” in mental health care, which is highly developed in somatic care (36, 57). This notion means that many psychiatric illnesses demonstrate a staged or continuum model of illness progression, including a palliative stage. Somewhat differently than in somatic care, one must leave room here for both positive developments of the illness and regression. Another factor is the relationship/connotation of “palliative psychiatry” with effective “hospice care” and forms of medically assisted dying. The concept “palliative care” is indeed used by various authors in the context of life-threatening conditions, such as refractory forms of anorexia nervosa (58, 59). In other publications, the “palliative care approach” is explicitly separated from the dying process (25, 37, 53). In both cases, there is a shared concern regarding harm reduction and alleviation of symptoms. A clear distinction between the various intersections between “end-of-life care” and mental health care, and a careful choice of names seems crucial here.

Finally, of course, the trade-off between doing good and not harming also appears in the context of active life-termination, which we discussed above (10, 23, 31, 47). Although a number of authors discuss this topic specifically targeting people with SPMI and deal with considerations about the legal framework, the estimation of autonomy and the difficult position of the person seeking help, the number of articles explicitly mentioning SPMI is rather limited compared to the body of literature dealing with MAID in persons with psychiatric suffering as a whole.

### The importance of virtues and attitudes

In addition to the central principles from bioethics discussed above, we find in the literature – although less prominent or structured way – that several virtues, attitudes and elements more commonly related to care-ethics, also have an important place in the development of good end-of-life care for persons with SPMI. In their publications, many authors refer to the importance of interpersonal relationships and the central position that fundamental attitudes and virtues occupy in caring for persons with SPMI (2, 11, 17, 19, 21, 28, 29, 60). A respectful attitude; taking responsibility; upholding human dignity; compassion; trust; love; honesty; non-abandonment; creativity; perseverance; taking time; being non-judgmental and remaining hopeful. All of these seem to be extremely important in order to achieve good, valuable care. Given the lack of a family network, the role and attitudes of care professionals as advocates and supporters in this context is crucial.

### Stakeholders in reflection and dialog

In the literature, relatively little attention is paid to the actors involved in ethical dialog or how this takes shape. It seems clear that the literature focuses mainly on the care professionals, with the doctor/psychiatrist, the nursing staff and other members of the multidisciplinary team being the most important stakeholders. In a few cases authors mention the role of the ethics committee or an ethical expert, in order to further explore a case and offer tools for dealing with moral stress (2, 19, 61). Only one of the consulted sources mentioned a specific methodology – a value-based deliberative tool, in the context of the development of an ethically grounded policy on medically assisted dying for persons with a psychiatric condition (62).

In addition to the care professionals, the patient’s family and relatives or the patient’s (legal) representative are mentioned in several places as important partners in ethical decision-making. From the literature we can conclude that involving the person with SPMI themselves in ethically charged decisions is often not self-evident in practice. Stigma, doubt about the ability to judge and difficulties in communication make participation difficult, although the voice of the person with SPMI is extremely important.

It is also striking that the role of policy, both at the level of the organization and at the level of wider society and politics, is relatively little addressed. When it does, it is mainly in taking responsibility for pointing out the unjust treatment of the target group (3, 37).

## Discussion

### Further reflections and lessons learned

It is striking that the majority of the selected sources for this study all mention the three central themes in end-of-life care for persons with SPMI: issues concerning the decision-making capacity of the person seeking care; access to care and stigma; and the possibility of a palliative care approach for this target group, related to the use of the concept of “futility.” These three themes can be nicely linked to the four principles of bioethics, as elaborated by Beauchamp and Childress: autonomy, justice, beneficence and non-maleficence (13). In addition, we see that the impact of care ethics is not omitted: fundamental attitudes and virtues seem at least as relevant as the core values in concrete care practice.

Although various authors explicitly link the three themes mentioned above to ethics and a value framework, many do not. The added value of this article may therefore be to specifically approach the care of persons with SPMI in general, and specifically with the focus on end-of-life care, as an ethical topic.

It is of course correct that the ethical themes and dilemmas that appear here, are not unique to the approach to persons with SPMI in this context. It is true, however, that in many ways the themes emerge more sharply or with greater complexity. Vulnerability, stigma, disruptive behavior, communication problems or doubts about decision-making capacity are elements we often encounter in (psychiatric) care. The specificity of this target group may be that these elements often occur in combination, or to a higher degree than in other target groups or in the general population.

On the other hand, the fact that the values, needs and desires in this target group are fundamentally the same as elsewhere in the population and in health care may also be an important element in the de-stigmatization of quality and good end-of-life care for persons with SPMI. We can also hypothesize that methodologies and forms of ethics support used elsewhere in health care can also be useful here. Other good practices in cross-sector training and education, first steps in developing a palliative care approach and existing tools and insights to support the decision-making capacities of vulnerable patients may also be helpful here. For example, it is important to recognize that making decisions is often not a purely rational consideration, but also includes emotional and relational elements, like the goals and leading values in one’s life (63). It is important to be aware that decision-making capacity is an ability that fluctuates in time and is often dependent on context, also in this target group. There are significant opportunities for good practices with regard to shared decision-making involving patients, care professionals and family (16).

This does not detract from the fact that we are faced with the social responsibility of adapting and tailoring the care of these persons to their needs, which often also show important similarities and even overlaps with the needs of other vulnerable target groups, such as homeless persons or persons with severe mental disabilities (29). The possibility of integrating elements of palliative care and its philosophical principles into different areas of (psychiatric) care, partly independent of the context of the end of life and its connotations, appears to be an important challenge and opportunity. Central to this is a better integration of care in all areas of life, a focus on quality of life and a flexible, creative approach to all elements of care. “Palliative care” should not be understood here as the loss of hope or abandonment of the patient, but rather as a form of care in which professionalism, quality and reflexivity are paramount (53).

Another field that poses important challenges is scientific research on this target group. The literature clearly shows that the misperceptions held by the general population and care professionals are often also reflected in researchers. The lack of scientific evidence also has a reinforcing effect on the stigmatization of the target group and an impact on the supply of care that is insufficiently geared to the needs of persons with SPMI. Conversely, involving persons with SPMI and their families can be an important factor in a fairer treatment and better adapted care, in which ethical considerations play a very central role.

### Limitations and strengths of the study

A first limitation of this study is that only peer-reviewed articles in English and Dutch were included. Therefore, we may miss evidence that is present in other languages, professional journals, handbooks or other local sources.

Although only peer reviewed articles published in academic journals were included and the articles were selected based on the independent judgment of two researchers with extensive expertise regarding care ethics and end-of-life decisions, no additional external quality check was performed. This may affect the quality of the sources included.

Another disadvantage is that there is no consensus definition of the concept “SPMI” as such, and other concepts may refer to the same population. As a result, it is possible that certain sources were not included in this review when authors referred to this target group under a different heading, such as the more familiar “SMI,” of which the group of persons with SPMI appears to be a subgroup, or when authors only mention a certain disorder, such as schizophrenia, without using the broader umbrella term “SPMI.” Also, sources which use the term irremediable psychiatric suffering, while referring to a subpopulation of the group of persons with SPMI, have been excluded. Despite the limitations of the search, a wide range of articles were nevertheless included, highlighting a wide variation both by underlying condition, end-of-life issues, geographic distribution and ethical aspects.

An important strength of this study is its specific focus, both on the target group and on the subject matter. To our knowledge, it is the first article that specifically addresses the ethical dilemmas in end-of-life care as a general theme for persons with SPMI.

### Conclusion

In this study, we sought to identify, by means of a scoping review of the literature, the main ethical dilemmas and issues regarding end-of-life care for persons with SPMI. We investigated which values, principles and virtues authors recalled to frame these issues, and looked at how ethical dialog was established and who participates in it. The four guiding principles of biomedical ethics – autonomy, justice, non-maleficence and beneficence – can be used to interpret the challenges in end-of-life care for persons with SPMI and to give an answer on our research questions: The first theme regards autonomy, and more specifically the assessment of (impaired) decision-making capacity of the persons in this target group. The balancing act between respecting the patient’s autonomy on the one hand and providing optimal care and preventing harm on the other, plays a central role here. This dilemma is even more pronounced when it comes to choices within the framework of medical assistance in dying. The role of representatives and family (and their absence) is also important. Opportunities for care are to be found in supporting and strengthening the voice of the person seeking care and in developing end-of-life skills in care professionals.

A second theme deals with the access of persons with SPMI to quality care, in particular palliative care and medically assisted aid in dying. In many cases, stigma and discrimination lead to higher thresholds to care, even though the needs and wishes are similar or even higher than those of the general population. Points of attention for a more equal and just treatment of the target group lie in the de-fragmentation of the care sectors, cross-sectoral cooperation and training, and forms of care that are better attuned to the concrete needs of the target group. The latter includes a call to take social responsibility to invest in care for special target groups, whereby low-threshold, community-based care initiatives close to the care recipient are given a chance to develop.

A third and final major theme is the possibility of developing a palliative care approach for persons with SPMI, and the more central role that the concept of “futility” plays in this. The use of this terminology presents several opportunities and challenges. Mental health care is first and foremost a setting that works toward safety, recovery, hope, and life perspective. A certain interpretation of the terms “palliative care” and “futility” seems to be completely at odds with this. Nevertheless, several authors argue for looking at the care of persons with SPMI from these concepts, with a view to increasing quality of life and well-being and reducing suffering. Good practices, careful elaboration of the concept of staging, more focused (qualitative) research and further clarification of concepts are the challenges here.

In addition to the core values, many authors also indicate, although in a more unstructured way, the importance of fundamental attitudes and virtues within the care relationship. Trust, non-abandonment, taking responsibility and compassion are some of the most important of these.

Furthermore, the literature gives us some insights into how ethical dialog is conducted and who participates in it. In addition to the team of care professionals, we see that family and legal representatives are often involved in ethical dilemmas that arise around the end of life, including refusing life-saving treatments. In several cases, the opinion of an ethics committee or the support of an ethics consultant could facilitate the elucidation of the dilemma and reduce moral stress. The literature also specifically indicates the importance of seeking ethical advice when developing scientific research. However, it is notable that the voice of the persons with SPMI themselves, although several authors stress its importance, is little heard. From this we learn that existing prejudices and stigma around this patient population are very real, and that mitigating these through additional awareness, training and other tailored care models are strongly recommended.

## Author contributions

LM was responsible for all steps of the research. AL assisted in the selection of source material and analysis. AL and MV critically reviewed the manuscript. All authors contributed to the article and approved the submitted version.

## Funding

This research was supported by the Special Research Fund (BOF) KU Leuven Grant number: ebc7b31d-c18d-4d94-9639-cfdb561abcdf.

## Conflict of interest

The authors declare that the research was conducted in the absence of any commercial or financial relationships that could be construed as a potential conflict of interest.

## Publisher’s note

All claims expressed in this article are solely those of the authors and do not necessarily represent those of their affiliated organizations, or those of the publisher, the editors and the reviewers. Any product that may be evaluated in this article, or claim that may be made by its manufacturer, is not guaranteed or endorsed by the publisher.
