# Supplementary material for: Mapping the ethical aspects in end-of-life care for persons with a severe and persistent mental illness: A scoping review of the literature
Source: Front Psychiatry. 2023 Mar 16;14:1094038. doi: 10.3389/fpsyt.2023.1094038 (PMC10062453; doi:10.3389/fpsyt.2023.1094038)
Supplement: Supplementary file 1 [file Table_1.DOCX]

Studies included in the analysis

N= 50

Excluded

Not relevant (n=2)

Excluded

Not relevant (n =3)

Excluded

Not relevant to the study

N= 255

Full text articles second opinion assessed (n=52)

**included**

**eligibility**

Full text articles assessed for eligibility

N= 55

Title and abstract review: 310

**screening**

Excluded

Duplicates: n =546

**Identification**

**Literature search**

**Electronic databases**

Scopus: n =283

Medline: n=234

Pubmed: n=226

Pubmed central: n=81

Atla: n=0

Psycharticles: n= 20

**Hand-searching: n=12**

Total: n=856
