# Supplementary material for: Mapping the ethical aspects in end-of-life care for persons with a severe and persistent mental illness: A scoping review of the literature
Source: Front Psychiatry. 2023 Mar 16;14:1094038. doi: 10.3389/fpsyt.2023.1094038 (PMC10062453; doi:10.3389/fpsyt.2023.1094038)
Supplement: Supplementary file 2 [file Table_2.DOCX]

**Annex 2: Summary of included articles and ethical aspects in End-of-life care for persons with SPMI**

| **number** | **Authors/year** | **Article type** | **Aims/description** | **Ethical aspects and themes** |
| --- | --- | --- | --- | --- |
| 1 | Trachsel  2018 | opinion | The ethical importance of assessing end-of-life care preferences in patients with severe and persistent metal illness | Risk of over-treatment  DMC is present  Do not express a wish for MAID more often  More research into palliative care approach |
| 2 | Stoll et al  2021 | Ethical argumentation | The way perceived burdensomeness influences the wish for hastened death in persons withs SPMI | Importance of DMC – Autonomy contingent to perceive burdensomeness.  Risk of stigma and paternalism. Need to ‘competent supported DM’  Access to MAID  Loss of dignity  Physician trained to prevent suicide  Training and research needed  Palliative approach as beneficial  Development of guidelines |
| 3 | Hodel et al  2019 | Original research | Survey regarding attitudes toward assisted suicide request in the context of SPMI in Swiss psychiatrists | Autonomy versus care (prevention of suicide)  Responsibility towards vulnerable persons (non-abandonment)  Need for guidelines regarding DMC  More research needed towards a palliative care approach and sedation |
| 4 | Trachsel et al  2019 | Original research | Survey regarding the acceptability of palliative care approaches for patients with SPMI in Swiss psychiatrists | Risk of association between palliative care and lose of hope  Balancing cure and care  A palliative approach does not exclude recovery  More research needed (ao a conceptual framework) |
| 5 | Butler et al  2018 | Retrospective study | Study regarding access to specialist palliative care for persons with SPMI | Access to specialist care is 3.5 times lower, needs and expectations are  the same  siloing of care and bad coordination of it  perceptions in healthcare workers regarding DMC  stigma  responsibility in offering continuity of care (society)  more research  nurse has an important role as facilitator and liaison |
| 6 | Carlsson et al  2017 | Critical review | Ethical and methodological issues in quantitative studies involving persons with SPMI | Access to being involved in qualitative research is hindered by  assumptions regarding vulnerability (protectionism)  Need to consult ethics commission  Upholding the principles declaration Helsinki  Importance of a balanced Informed Consent  Involving patients in research  More research could mean less stigma |
| 7 | Decorte et al  2020 | Opinion/case report | Sketching a palliative care model for persons with SPMI: ‘Oyster care’ | Unfair treatment of a vulnerable group  Need of a palliative approach, causing less harm – doing good  Dynamics regarding autonomy and coercion  Dynamics regarding MAID  Need for more research and societal responsibility  Role of care professionals as ‘substitute family members’ |
| 8 | Dierickx et al  2017 | Retrospective study | Analysis of officially reported cases of euthanasia for persons with psychiatric disorders or dementia in Belgium | Assessment of DMC  Vulnerability  Importance of accessibility of MAID  Need for quality guidelines |
| 9 | Trachsel et al  2016 | opinion | Definition, scope, benefits and risks regarding palliative psychiatry for persons with SPMI | A palliative approach can alleviate suffering and improve QOL  Debate regarding futility necessary  A palliative approach is not opposite to recovery  Negative connotations with EOL  Controversy regarding palliative sedation and positioning toward MAID  Impaired DMC as a barrier  More research needed |
| 10 | Baruth et al  2021 | Systematic review | End-of-life care in schizophrenia | Positioning of a palliative approach  DMC/autonomy: role of pathology in decision-making?  Refusal of life sustaining treatment  Need for ethical analysis in DM process  Coercion and the role of the guardian/surrogate decision-maker  Negative behavior and communication-problems limit access to care  Stigma  Importance of ACP  Role of ethics commission and ethical expert  Importance of early detection and training to limit disparity  Virtues: being compassionate, having patience |
| 11 | Roberts et al  2004 | Ethical analysis | Ethical use of long-acting medications in treatment of SPMI | Autonomy: DMC and doubt about ‘authentic choice’  Role of coercion: how to do no harm, do good and be just? |
| 12 | Deodohar  2016 | Retrospective study | Current trends and future directions in end-of-life care and psychiatry in India | Principles of a ‘good death’: dignity, privacy and spiritual support  Four principles of biomedical ethics are essential in EOL care  Need for ACP  Futility debate  Psychiatrist as ethical expert  Chances for palliative psychiatry  Access to palliative care is limited |
| 13 | Levitt et al  2020 | opinion | Reflections regarding the concept of futility in psychiatry | Do no harm: prevention of therapeutic stubbornness  Doing justice to autonomy: give patients a voice,  against ‘beneficent paternalism’  Beneficence: improving QOL  Importance of staging in palliative psychiatry  Futility does not equal terminal or loss of hope. There is room for recovery  Need of safe places to dialogue |
| 14 | Lopez-Castroman  2017 | Opinion | Reflections regarding the practice of psychiatric euthanasia | MAID in psychiatry as controversial theme  Importance of assessing DMC  Palliative psychiatry as alternative |
| 15 | Forrster et al  2006 | Qualitative study | Ethico-legal issues in relation to end-of-life care and institutional mental health in Queensland, Australia | Perceived caregiver dilemma: providing death with dignity versus risk of legal pursuit  Autonomy/DMC related issue: ACP not possible because authentic informed consent is not possible  Need for good education in care professionals so to better understand EOL legal issues  Some care professionals perceive not providing reanimation as ‘death making’ |
| 16 | Lindblad et al  2019 | opinion | Reflections regarding the idea of a palliative care approach in psychiatry | A separate definition might lead to more stigma  Palliative care has a connotation with the terminal faze  What is the relation between a palliative care approach and MAID?  Need for more research and practice-based examples |
| 17 | Verhofstadt et al  2020 | survey | Belgian psychiatrists’ attitudes towards, and readiness to engage in euthanasia assessment procedures with adults with psychiatric disorders | Psychiatrists may have moral reasons not to participate in the euthanasia assessment procedure  Reference to the general ethical debate regarding euthanasia in this context  Questions regarding the accessibility of MAID for the target group  More research needed regarding values, norms and religious aspects in euthanasia and psychiatric suffering |
| 18 | Harman  2017 | Retrospective study | Psychiatric and palliative care in the Intensive care unit | DMC is complex in the SPMI target group. This affects ACP  Surrogate decision-makers and guardians have an important role (if they can be found)  Palliative care needs to compensate for a lack of ACP, DMC and social network |
| 19 | Ebenau et al  2020 | Qualitative study | Experiences of healthcare professionals, volunteers and experts-by-experience, of Palliative care for persons with substance use disorder and multiple problems in the Netherlands | Stigma  Virtue: building a relationship of trust  Siloing in care, bad coordination, limited resources and late detection  Need of consultation, training and creativity  Autonomy/DMC: patients sometimes refuse life-saving treatment  Dilemma: Access to pain medication in persons know for substance-abuse?  Virtue: responsibility (especially incases with limited social network  Virtues: alert, active, taking time, respect, non-judgmental, non-hierarchical  Access to care: homelessness |
| 20 | Ebenau et al  2018 | methodological | A study protocol regarding palliative care for persons with a substance use disorder and multiple problems | Homelessness and limited social network  Strong autonomy regarding EOLD decisions, but limited communication  Methodological: time to decide, IC, anonymity, experts by experience consulted, tailored interviews (time-management) |
| 21 | Torres-Gonzalez et al  2014 | review | Unmet needs in the management of schizophrenia | Stigma  Importance of respecting autonomy  Justice: relation between unmet needs and suicide/lower life expectancy |
| 22 | Shalev et al  2017 | editorial | End-of-life care in patients with severe mental illness | Limited access to palliative care as ethical dilemma  Access is limited by: siloing, limited resources (care models now are expensive and offer limited QOL), stigma and perceptions of DMC, lack of expertise and training  Opportunities lie in the use of technology, a multidisciplinary approach, palliative approach and community-based models |
| 23 | Kirby  2019 | Original research | Two models to balance competing interests and obligations in metal healthcare practice and policy | Value-based deliberative tool  Using moral relational space (power free) in therapeutic engagement |
| 24 | West et al  2020 | Scoping review | Exploring the end-of-life needs of Homeless Persons and Barriers to appropriate care | Dilemma: autonomy versus care  Paternalism with regard to ACP  Virtues: respect, dignity, responsibility  Access to palliative care limited by: -stigma and discrimination  -Noncompliance and disturbing behavior  -Drug seeking behavior  -limited social network  -siloing in care and insufficient specialization towards the target group  Good practices: enhancing ACP, avoiding ‘paternalistic’ hospital setting (connotation of coercion), providing care where the patient actually lives |
| 25 | Strand et al  2020 | opinion | Reflection on and clinical implications of a palliative care approach in psychiatry | A palliative care approach can contribute to the non-maleficence and beneficence principles  Questions regarding staging  Importance of shared decision-making and patient centeredness  Negative connotation with EOL  Relation with regard to recovery  Importance of an ethical/value-bases care approach |
| 26 | Etgen  2020 | Retrospective study | Care series of introducing palliative care consultation in psychiatry | Possible alternative for the existing limited access to palliative care consultation |
| 27 | Grassi et al  2020 | Narrative review | Problems and solutions regarding cancer and severe mental illness | Limited access to care, social isolation  Stigma (prejudice about patients being unmanageable)  attitudes and virtues: dignity, hope, respect, non-abandonment  Problems related to DMC and surrogate decision-makers |
| 28 | Lemon et al  2016 | Survey/pilot study | Trust and the dilemmas of suicide risk assessment in non-government metal health services in Australia | Key value: trust (towards the client, tools and colleagues)  Important role of training and professionalism |
| 29 | Terpstra et al  2014 | Case report | Palliative care for terminally ill individuals with schizophrenia | Access to care palliative care is limited due to lack of insurance, different experience of pain in persons with SPMI, safety-issues, smoking  Difficulties regarding DMC and communication  Need of better collaboration and sharing of experiences between care professionals  Role of care professionals as ‘substitute family members’  Importance of death with dignity |
| 30 | Terpstra et al  2012 | opinion | Hospice and palliative care for terminally ill individuals with serious and persistent mental illness | Barriers to care are manyfold, f.i. Anxiety in care professionals to bring up the topic of EOL and stigma  Central role of DMC  Good practice: cross training among care professionals in mental healthcare and palliative care  Attitudes: compassion, person-centeredness |
| 31 | Irwin et al  2014 | review | Cancer care for individuals with schizophrenia | Central role of DMC  Prejudice regarding vulnerability  Stigma  Disparities (insurance, self-stigma, exclusion from research, communication-issues, homelessness)  Virtues: responsibility, trust  Importance of clarity in guidelines and the division of tasks in EOL-care |
| 32 | Guidry-Grimes  2019 | Case report | A case report regarding a very ill, homeless and psychiatrically complex person, told from the perspective of an ethics counselor | The clients fall between the ‘mazes of the net’ in every aspect: socially, legally, imparities regarding access to quality care  Autonomy/DMC related dilemma’s: absence of surrogate decision maker or family, refusal of life sustaining treatment, risk of paternalism  Ethical advisor as an ambassador between several institutions and as someone with a unique point of view. Can give support in cases of moral distress  Risk of being ‘given up’: cure versus care debate + limits to care?  No ACP  Stigma by care professionals (different attitude towards fi elderly patients)  Siloing  Wrong use of ‘comfort care’ (giving up on patient)  Core Values and attitudes: humility, creativity, trust, responsibility  Importance of a multidisciplinary approach to care |
| 33 | Roberts et al  2015 | Survey/pilot | Attunement and alignment of persons with schizophrenia and their preferred alternative decision-makers regarding treatment and research decisions | Individuals generally make decisions according to their values (autonomy, justice, non-maleficence and beneficence)  Central role of assessing DMC and the role of the guardian  Decisions of guardians and patients are mostly aligned |
| 34 | Nisavic et al  2019 | Case report | A 27 year-old woman with opiod use disorder and suicidal ideation | Dilemma: how to assess care and pain medication is someone with drug seeking behavior?  Values: trust, do no harm |
| 35 | Donald et al  2019 | Scoping review | Palliative care needs for persons with SPMI | The voice of the client is rarely heard, due to a lack of research and perceptions about autonomy and DMC  Siloing of care and lack of specialization results in limited access  Advantages of a palliative care approach and cross-training  The importance of care professionals as advocates  Importance of training in care professionals to do EOL conversations |
| 36 | Le Melle et al  2005 | Case report | Heart transplant in a young man with schizophrenia | Stigma and discrimination based on diagnosis of SPMI  Access to care limited/denied  Wrong use of ‘comfort care’ (giving up on patient)  Importance of the healthcare team and social network  Important value: trust  Dilemma: DMC in ACP  Role of ethics commission in resolving the case |
| 37 | Weimand et al | Descriptive study | A Qualitative/phenomenographic study regarding life sharing experiences of relatives of persons with severe mental illness | Key values and attitudes: trust, love, compassion, responsibility  Ethical dilemma: autonomy versus coercion when confronted with suicidality and aggression |
| 38 | Gloecker et al  2021 | Pilot survey | Nurse’s view on palliative care for those diagnosed with SPMI in Switzerland | Little support for coercion, in favor of QOL  Autonomy as central value – sometimes dilemmas arise with regard to optimal care/beneficence  Palliative care approach as an ethical alternative: cure (harm) versus care |
| 39 | Coulter et al  2021 | opinion | Evaluating ineffective treatments: a proposed model for discussing futility in psychiatric illness | Futility in psychiatry is an ethical problem, balancing autonomy, beneficence and non-maleficence  Palliative psychiatry as valid alternative  Decisions should be made with regard to quality of life, rather than based on than DMC |
| 40 | Stoll et al  2021 | Survey | Attitudes among Swiss psychiatrists with regard to compulsory interventions in SPMI and EOL situations | Dilemma: autonomy versus paternalism  Beneficence and benefice as crucial to the psychiatrist’s role  Importance of ethics support  DMC as important dilemma  legislation |
| 41 | Knippenberg et al  2020 | Qualitative study | Patient semi-structured interviews with regard to severe mental illness and palliative care | Little research has been done  Difficulties: communication, insight, medication, negative experiences with care professionals  Importance of ethical standards in research  Access to care limited: patients are reluctant to discuss EOL topics  Important role of trusted care professionals |
| 42 | Scolan et al  2013 | Case study | Ethical dilemma of the therapeutic decisions in the care for a patient with severe anorexia nervosa | Refusal of life sustaining treatment  Balancing autonomy, non-maleficence and beneficence  Crucial role of DMC  legislation |
| 43 | Lopez et al  2009 | Case report | Medical futility in psychiatry: palliative care and hospice care as a last resort in the treatment of refractory anorexia nervosa | Need for ethics support in difficult cases  Palliative care is a good alternative in some situations with regard to QOL  DMC as a central dilemma  legislation |
| 44 | Taylor et al  2018 | Case report | A clinical ethics case report on a case of refractory schizophrenia, attempted suicide and withdrawal of life support | Plead to take seriously the mental suffering and the wish for ACP  Role of ethics commission in supporting healthcare workers in difficult EOL cases |
| 45 | Moonen et al  2015 | Qualitative study | Experiences of psychiatric nurses in Flanders (Belgium) in dealing with existential suffering of patients with SPMI | Important values: autonomy, trust  Risk of paternalistic approach  Need for more research  Importance of the development of a palliative care approach  Context of euthanasia and the ethical debate regarding it |
| 46 | Elie et al  2017 | Survey | A comparative cross-sectional study regarding EOL care preferences in patients with SPMI and chronic medical conditions | Importance of palliative psychiatry with focus on QOL  Assessment of Autonomy and DMC in the context of treatment refusal  More research needed on ethical themes in this target group  Persons with SPMI have no negative attitude towards MAID |
| 47 | Den Boer et al  2019 | Systematic review | A systematic review in palliative care tools and interventions for persons with severe mental illness | Very little research regarding the target group  Conversations about EOL with persons with SPMI are possible should be encouraged  Access to somatic care is limited due to siloing of care and difficulties in communication |
| 48 | MCGorry et al  2012 | opinion | Commentary on ‘palliative models of care for later stages of mental disorder: maximizing recovery, maintaining hope and building morale | Importance of a staging model that has room for upward and downward movement  Negative connotations with regard to the concept ‘palliative care’  Siloing of care |
| 49 | Berk et al  2012 | Literature review | Palliative models of care for later stages of mental disorder | Importance of a staging model in SPMI  Palliative care approach as beneficial to QOL  Risk of doing harm (over-treatment)  Stigma towards persons with SPMI |
| 50 | Woods et al  2008 | Systematic review | Palliative care for persons with SPMI | Very little research available  DMC as central theme  Key values and attitudes: respect, truthfulness, dignity, hope, non-abandonment, compassion, power  ACP not self-evident  Access to care restricted  Lack of social network, role of substitute decision-makers  Treatment refusal |
