# Supplementary material for: Mapping the ethical aspects in end-of-life care for persons with a severe and persistent mental illness: A scoping review of the literature
Source: Front Psychiatry. 2023 Mar 16;14:1094038. doi: 10.3389/fpsyt.2023.1094038 (PMC10062453; doi:10.3389/fpsyt.2023.1094038)
Supplement: Supplementary file 3 [file Table_3.DOCX]

**Appendix 1: Conceptual framework of (treatment) decisions regarding End-of-life Care in and outside the Medical Context**

| **Medical Assistance in Dying (MAID)** |
| --- |
| **Active termination of life** |
| **Euthanasia** The act of a clinician intentionally ending the life of an individual by administering life ending/lethal drugs, upon the individual’s explicit request  **Physician-Assisted Suicide (PAS)**  The act of a clinician intentionally helping the individual to hasten death by providing or prescribing the individual with lethal medication, that the individual self-administers  **Active ending of life without explicit patient request** The act of administering drugs with the explicit intention of hastening death, in the absence of the individual’s request (non-voluntary) or against the individual’s will (involuntary). |
| **(Forgoing) Curative and/or life-sustaining treatment** |
| **Starting or continuing curative or life-extending treatment**  **Refusal of treatment**  The cessation or failure to provide curative or life-extending treatment because the patient refuses  **Non-Treatment Decision (DNR)**  The withholding or withdrawing of treatment, knowing that this may or will hasten the patient’s death:  1) Withholding treatment (sometimes called passive euthanasia)  the physician allows the individual to die by withholding life-prolonging treatment when believing that this serves the individual’s best interest and/or because the individual competently refused life-prolonging treatment  2) Withdrawing treatment (also referred to as passive euthanasia)  the physician allows the individual to die by withdrawing life-prolonging treatment when believing that this serves the individual’s best interest and/or because the individual competently refused the continuation of the life-prolonging treatment  3) By allowing the individual to Voluntarily Stop Eating and Drinking and hence the refusal of all food and liquids, including those taken through a feeding tube. |
| **Pain and symptom control** |
| **Palliative Care**  Specialized medical care for individuals suffering from a serious illness. This type of care is focused on the alleviation of suffering due to, e.g., symptoms or the distress surrounding illness, aiming to improve quality of life for both the patient and the family, based on the needs of the patient, not on the patient’s prognosis.  **Palliative Sedation**  The most far-reaching form of intensified pain and symptom alleviation at the end of life is intermittent or continuous deep sedation until death: the act of using medications intended to induce a state of decreased awareness or actual unconsciousness to relieve the burden of otherwise intractable suffering.  **Palliative Psychiatry (PP)**  An approach that improves the quality of life of patients and their families in facing the problems associated with life-threatening severe persistent mental illness (SPMI), e.g. refractory anorexia nervosa, through the prevention and relief of suffering by means of a timely assessment and treatment of associated physical, mental, social, and spiritual needs. PP focuses on harm reduction and on avoidance of burdensome psychiatric interventions with questionable impact.  **A Palliative Care Approach (PCA): Living with Severe and Persistent Mental Illness**  A model of care, e.g. Oyster Care, designed for persons with severe and persistent mental illness, based on elements of palliative philosophy. This approach may be appropriate if other, recovery-oriented treatment options have no (lasting) effect on the person's quality of life. Based on the futility of other treatment options with often serious side effects, a different approach in which quality of life, symptom control, therapeutic presence, and a thorough holistic and creative approach are key. A palliative care approach can be a stage within a continuum of care with different phases, within which upscaling and downscaling can occur. |
| **Dying without the assistance of physicians** |
| **Suicide** The individual intentionally hastens her death  **Assisted Suicide** A non-physician helps the individual to hasten her death |
